# Society Meetings

**Published:** 1893-03

**Authors:** 


### Society Meetings.

The next meeting of the Buffalo Microscopical Club will be held Monday evening, March 13, 1893. The evening will be devoted to a manufacturers' exhibition of microscopes and accessories. The following firms have accepted and will take part: Spencer & Smith, Buffalo; Queen & Co., Philadelphia; Joseph Zentmayer, Philadelphia; Bausch & Lomb, Rochester; Gundlach Optical Co., Rochester; E. H. Griffith, Rochester. A reduction from catalogue prices will be granted members of the club and physicians on all orders placed with these firms during the evening.

---

The next stated meeting of the Buffalo Academy of Medicine will be held Tuesday evening, March 21, 1893. The Section on Anatomy, Physiology, and Pathology will have charge of the programme.

---

Dr. George M. Sternberg, Deputy Surgeon-General U. S. Army, Executive President of the Pan-American Medical Congress, Section of Military Medicine and Surgery, announces that the following gentlemen have been duly appointed members of the Advisory Council of this section: Colonel Louis Read, M. D., Surgeon-General N. G., Pa.; Newton L. Bates, M. D., Medical Director U. S. N.; J. R. Tryon, M. D., Medical Inspector, U. S. N.; Lieut.-Colonel Eustathius Chancellor, M. D., Medical Director, N. G., Mo.; Brevet Lieut.-Col. A. A. Woodhull, M. D., Surgeon, U .S. A.; Maj. Joseph H. Corson, M. D., Surgeon, U. S. A.; Maj. George Henderson, M. D., Medical Director, N. G., D.C.; C. N. Hoagland, M. D., ex-Surgeon, Ohio Vols.; Bedford Brown, M. D., ex-Surgeon, C. S. A.; H. C. Goodman, M. D., ex-Surgeon, U. S. Vols.; Melancthon Storrs, M. D., ex-Surgeon, Conn. Vols.; O. D. Ball, M. D., Pension ex-Surgeon, Albany, N. Y.; Capt. H. O. Perley, M. D., Assistant Surgeon, U. S. A.

---

Dr. Henry S. Upson, of Cleveland, O., delivered an interesting lecture before the Buffalo Microscopical Club, at its last meeting, on A Few Remarks on Nerve Cells and Their Mode of Action.

---

The Section on Surgery of the Buffalo Academy of Medicine has elected the following officers for the ensuing year: President, H. E. Hayd, M. D.; Vice-President, M. Hartwig, M. D.; Secretary and Treasurer, C. A. Ring, M. D.

Among the Buffalo physicians who attended the recent meeting of the New York State Medical Society, held at Albany, were Drs. Daggett, Hopkins, Mynter, Hayd, Park, Stockton, Bergtold, Thornton, Howe, and Krauss. Dr. Lapp, of Clarence, was also present. Papers were read by Dr. Wm. C. Krauss on Reflex Disturbances in the Causation of Epilepsy; by Dr. Roswell Park on The Etiology of Carcinoma, and by Dr. Chas. G. Stockton on The Disturbances of the Motor Function of the Stomach—their Diagnosis, Symptoms, and Treatment. Dr. H. Mynter participated in the discussion on Tuberculous Disease of the Bones and Joints.

---

The next meeting of the Section on Surgery of the Buffalo Academy of Medicine will be held Tuesday evening, March 7, 1893. The programme for the evening will be: The Causes and Theories of Syphilis, by Ernest Wende, M. D.; Syphilis and Marriage, by C. C. Frederick, M. D.; The Treatment of Syphilis, by William H. Heath, M. D.

---

The next meeting of the Section on Medicine of the Buffalo Academy of Medicine will be held at the Academy rooms, Tuesday evening, March 14, 1893. The programme will be devoted to reports of clinical cases by Dr. E. A. Smith and others.

